# Barrieren des Fahrradfahrens im Alltag: Ergebnisse der bevölkerungsweiten Befragungsstudie „Gesundheit in Deutschland“

**DOI:** 10.1007/s00103-026-04311-w

**Published:** 2026-10-08

**Authors:** Kristin Manz, Susanne Krug, Gert B. M. Mensink, Julika Loss

**Affiliations:** Abteilung für Epidemiologie und Gesundheitsmonitoring, Robert Koch-Institut, Nordufer 20, 13353 Berlin, Deutschland

**Keywords:** Radfahren, Aktive Mobilität, Hinderungsgründe, Bewegung, Gesundheitsförderung, Cycling, Active transport, Obstacles, Physical activity, Health promotion

## Abstract

**Hintergrund:**

Das Zurücklegen alltäglicher Wege mit dem Fahrrad anstelle des Autos hat vielfältige positive Effekte auf die individuelle und öffentliche Gesundheit. Da der Anteil der mit dem Fahrrad zurückgelegten Wege in Deutschland derzeit gering ist, werden in diesem Beitrag Barrieren des alltäglichen Fahrradfahrens und Unterschiede zwischen soziodemografischen Gruppen untersucht.

**Methoden:**

Im Rahmen der Jahreserhebung 2025 des bevölkerungsbezogenen Panels „Gesundheit in Deutschland“ des Robert Koch-Instituts wurden Erwachsene zur Nutzung des Fahrrads im Alltag und zu Barrieren des Fahrradfahrens befragt. Unterschiede zwischen soziodemografischen Gruppen wurden mittels Regressionsanalysen untersucht. In die Analysen gingen Daten von 32.374 Personen ein.

**Ergebnisse:**

Die 5 am häufigsten ausgewählten Barrieren waren schlechte Wetterbedingungen (51,1 %), zu lange Wege (45,1 %), nicht verschwitzt am Zielort ankommen zu wollen (42,8 %), der Transport von Gepäck bzw. Personen (34,4 %) und fehlende Umkleiden und Duschen (31,9 %). Zwischen den soziodemografischen Gruppen zeigten sich signifikante Unterschiede: Für Frauen waren sicherheitsbezogene Barrieren relevanter, für ältere Personen und Personen mit formal niedriger Bildung die eigene Gesundheit und Fitness sowie das Fehlen eines (guten) Fahrrads. Personen in ländlichen Wohnregionen nannten häufiger zu lange und zu bergige Wege.

**Diskussion:**

Die Ergebnisse zeigen, dass Barrieren des Fahrradfahrens sowohl auf umweltbezogener als auch auf individueller Ebene bestehen und daher Maßnahmen auf mehreren Ebenen erforderlich sind. Insbesondere sollten die Bedarfe von Bevölkerungsgruppen adressiert werden, die das Fahrrad bislang seltener nutzten, um Barrieren abzubauen und die gesundheitliche Chancengleichheit zu fördern.

**Zusatzmaterial online:**

Zusätzliche Informationen sind in der Online-Version dieses Artikels (https://doi.org/10.1007/s00103-026-04311-w) enthalten.

## Abstract (en)

**Background:**

Traveling by bicycle rather than by car has a wide range of positive effects on individual and public health. As the proportion of journeys made by bicycle in Germany is currently low, this article examines the barriers to everyday cycling and differences between socio-demographic groups.

**Methods:**

As part of the 2025 annual survey of the Robert Koch Institute’s population-based panel “Health in Germany,” adults were asked about their everyday use of bicycles and the barriers to cycling. Differences between socio-demographic groups were examined using regression analyses. Data from 32,374 individuals were included in the analyses.

**Results:**

The five most frequently cited barriers were poor weather conditions (51.1%), distances being too long (45.1%), not wanting to arrive at the destination sweaty (42.8%), transporting luggage or passengers (34.4%), and a lack of changing rooms and showers (31.9%). Significant differences were observed between socio-demographic groups: safety-related barriers were more relevant for women, whilst for older people and those with a lower level of formal education, their own health and fitness, as well as the lack of a (good) bicycle, were more significant. People living in rural areas were more likely to cite journeys that were too long or too hilly.

**Discussion:**

The results show that barriers to cycling exist at both the environmental and individual levels, and therefore measures are required at multiple levels. In particular, the needs of population groups who have so far used bicycles less frequently should be addressed to reduce barriers and promote health equity.

## Einleitung

Die Wahl des Verkehrsmittels im Alltag kann die Gesundheit auf unterschiedliche Weise beeinflussen. Insbesondere der Umstieg vom motorisierten Individualverkehr (z. B. Pkw, motorisierte Zweiräder) auf aktive Mobilität bietet gesundheitliche Potenziale. Mit aktiver Mobilität ist in diesem Zusammenhang das Zurücklegen von Wegstrecken durch die eigene Muskelkraft gemeint, etwa zu Fuß, mit dem Fahrrad oder Tretroller. Ein wesentlicher gesundheitlicher Nutzen aktiver Mobilität liegt in der höheren körperlichen Aktivität im Vergleich zum motorisierten Individualverkehr. Die positiven Effekte von Bewegung, insbesondere für die Prävention nichtübertragbarer Erkrankungen, sind umfassend belegt [1, 2].

Studien zur aktiven Mobilität zeigen, dass sie das Risiko für kardiovaskuläre Erkrankungen, chronische Erkrankungen wie Diabetes mellitus Typ 2, ausgewählte Krebsarten und vorzeitige Sterblichkeit senken kann [3]. Dies gilt insbesondere für das Fahrradfahren, das im Vergleich zum Gehen häufiger mit einer höheren Bewegungsintensität einhergeht [4]. Auch das Fahren von Pedelecs (Fahrrädern mit elektrischer Unterstützung) ist mit positiven Gesundheitseffekten verbunden [5]. Zudem kann sich aktive Mobilität förderlich auf die psychische Gesundheit auswirken [6]. Besonders groß ist der gesundheitliche Nutzen, wenn zuvor körperlich inaktive Personen auf aktive Mobilität umsteigen [7].

Neben den positiven gesundheitlichen Effekten kann aktive Mobilität auch mit gesundheitlichen Risiken verbunden sein, etwa durch Verkehrsunfälle oder eine erhöhte Exposition gegenüber Luftschadstoffen. Studien zeigen jedoch, dass der gesundheitliche Nutzen aktiver Mobilität die gesundheitlichen Risiken deutlich überwiegt [7, 8].

Zusätzlich zu den positiven Effekten für die individuelle Gesundheit ergeben sich auch Vorteile für die Gesundheit der Bevölkerung, wenn motorisierter Individualverkehr durch aktive Mobilität ersetzt wird [9]. So kann eine Verringerung des Autoverkehrs sowohl die Luftqualität verbessern, indem die verkehrsbedingte Belastung durch Feinstaub und Stickoxide sinkt, als auch die Lärmbelastung reduzieren [9]. Weitere Co-Benefits aktiver Mobilität sind der Beitrag zum Klimaschutz durch geringere Emissionen [10] sowie eine Reduktion von Gesundheitsausgaben [11].

Die Verkehrswende eröffnet somit die Chance, Lebenswelten gesundheitsförderlicher zu gestalten und zur Gesundheit der Bevölkerung beizutragen. Von den unterschiedlichen Formen der aktiven Mobilität hat das Fahrradfahren das größte Potenzial, den motorisierten Individualverkehr zu ersetzen, da mit dem Fahrrad im Vergleich zum Gehen häufig größere Distanzen zurückgelegt werden. Die zunehmende Nutzung von Pedelecs in der Bevölkerung kann hierzu weiter beitragen.

Nach den Ergebnissen der Studie „Mobilität in Deutschland“ aus dem Jahr 2025, einer Erhebung im Auftrag des Bundesministeriums für Verkehr (BMV), betrug der Anteil aller mit dem Fahrrad zurückgelegten Wege in Deutschland 11 % [12]. Demgegenüber fiel ein Anteil von 53 % auf den motorisierten Individualverkehr, was bedeutet, dass mindestens jeder zweite Weg mit dem Auto oder einem motorisierten Zweirad zurückgelegt wurde. Die Studie „Gesundheit in Deutschland aktuell“ (GEDA) aus den Jahren 2019/2020 hat gezeigt, dass lediglich 26 % der Erwachsenen in Deutschland mindestens eine Stunde pro Woche Wegstrecken mit dem Fahrrad zurücklegten [13]. Seltener nutzten Frauen, ältere Menschen und Personen mit niedriger Bildung das Fahrrad für alltägliche Wegstrecken.

Angesichts der gesundheitlichen Vorteile des Fahrradfahrens stellt sich die Frage, weshalb eine Vielzahl an Personen nicht oder nur in geringem Umfang das Fahrrad als Verkehrsmittel nutzt. In der Literatur werden unterschiedliche Einflussfaktoren auf die aktive Mobilität diskutiert, die sich nach sozialökologischen Modellen den nachfolgenden Bereichen zuordnen lassen [14, 15]:

- Individuum (soziodemografische Faktoren, Einstellungen),
- soziokulturelle Umwelt (soziale und kulturelle Normen, Freunde, Familie),
- bebaute Umwelt (Infrastruktur, Sicherheit, Attraktivität) und
- politische Rahmenbedingungen (Richtlinien, Regulierungen).

Internationale Studien zeigen überwiegend einen Einfluss der bebauten Umwelt auf die aktive Mobilität, während soziokulturelle Faktoren bisher nur wenig untersucht wurden [14, 16].

Für die Konzeption zielgerichteter Maßnahmen zur Förderung des Fahrradfahrens ist es wichtig, mögliche Barrieren zu kennen und Unterschiede zwischen soziodemografischen Gruppen zu berücksichtigen. Von besonderer Bedeutung sind dabei Kenntnisse zu den Hinderungsgründen von Personen, die das Fahrrad bislang wenig für ihre alltägliche Mobilität nutzen, da allgemeine Maßnahmen zur Förderung des Fahrradfahrens nicht zwangsläufig dazu führen, dass auch die Diversität der Fahrradfahrenden zunimmt [17, 18].

In der vom BMV beauftragten Studie „Fahrrad-Monitor 2025“ wurden 14- bis 69-Jährige, die nie oder selten mit dem Fahrrad fuhren, nach den Gründen für die seltene Fahrradnutzung gefragt [19]. Am häufigsten wurden das Wetter und zu weite Strecken genannt. Auf Unterschiede nach soziodemografischen Faktoren wurde nicht eingegangen.

Bislang gibt es kaum Studien, die subjektiv empfundene Barrieren des Fahrradfahrens als Form der aktiven Mobilität über die gesamte erwachsene Bevölkerung in Deutschland hinweg erfassen und Unterschiede zwischen soziodemografischen Gruppen analysieren. Die vorliegende Auswertung geht daher folgenden Fragestellungen nach:

1. Welche Barrieren des Fahrradfahrens als Form der aktiven Mobilität werden von Erwachsenen in Deutschland subjektiv wahrgenommen?
2. Inwiefern unterscheidet sich die Relevanz dieser Barrieren zwischen soziodemografischen Gruppen sowie zwischen ländlichen und städtischen Wohnregionen?

## Methoden

Die vorliegende Auswertung basiert auf Befragungsdaten der Jahreserhebung 2025 des bevölkerungsbasierten Panels „Gesundheit in Deutschland“ des Robert Koch-Instituts. Detaillierte Informationen zur Rekrutierung, zur Rekrutierungsbefragung und zur Stichprobe wurden an anderer Stelle publiziert [20]. Um saisonale Effekte zu berücksichtigen, wurden die registrierten Panelteilnehmenden zwischen Januar und Dezember 2025 in 4 Teilwellen, d. h. einmal pro Quartal, schriftlich befragt. Sie konnten zwischen einem Onlinefragebogen (Computer-Assisted Web Interviewing, CAWI) und einem papierbasierten Fragebogen (Paper-and-Pencil Interviewing, PAPI) wählen. Die Responsequote, definiert als Anteil der Teilnehmenden im Verhältnis zu den im Panel registrierten Personen, lag in den einzelnen Teilwellen gemäß den Standards der American Association for Public Opinion Research (AAPOR) zwischen 67 % und 76 % [21].

In die Analysen wurden Daten von 32.374 registrierten Panelteilnehmenden ab 18 Jahren einbezogen, die Angaben im Fragebogen C zum Fahrradfahren gemacht haben (Teilwelle 1: *n* = 8588, Teilwelle 2: *n* = 8226, Teilwelle 3: *n* = 7813, Teilwelle 4: *n* = 7747).

### Erfassung der Variablen

#### Soziodemografische Variablen

In die Analysen der soziodemografischen Unterschiede wurde die geschlechtliche Identität („weiblich“, „männlich“, „offene“ Antwortoption) einbezogen, wobei nur Personen berücksichtigt wurden, die sich als weiblich oder männlich identifizierten. Die Bildungsgruppen („niedrig“, „mittel“, „hoch“) wurden anhand der Angaben zum höchsten allgemeinbildenden Schulabschluss sowie zum höchsten beruflichen Ausbildungs- bzw. (Fach‑)Hochschulabschluss nach der CASMIN(Comparative Analysis of Social Mobility in Industrial Nations)-Klassifikation gebildet [22]. Zur Einordnung des Wohnortes wurde die politische Gemeindegrößenklasse mit folgenden 4 Kategorien herangezogen: „ländlich (< 5000 Einwohnende)“, „kleinstädtisch (5000 bis < 20.000)“, „mittelstädtisch (20.000 bis < 100.000)“ und „großstädtisch (≥ 100.000)“.

#### Variablen zum Fahrradfahren

Die Teilnehmenden wurden, bezogen auf eine typische Woche in den letzten 4 Wochen, nach der Anzahl der Tage pro Woche gefragt, an denen sie ihre Wegstrecke mit dem Fahrrad zurücklegten. Hierbei sollten auch Fahrten mit einem Fahrrad mit elektrischer Unterstützung eingeschlossen werden. Die Antwortkategorien lauteten: „An 6–7 Tagen“ (1), „An 4–5 Tagen“ (2), „An 1–3 Tagen“ (3), „Seltener als an einem Tag“ pro „Woche“ (4) oder „Nie“ (5). Für die Analysen wurden die Kategorien 1 und 2 zur Kategorie „An mindestens 4 Tagen pro Woche“ zusammengefasst. Der zeitliche Bezug dieser Variable „Tage pro Woche in den letzten vier Wochen vor der Befragung“ wird zur besseren Lesbarkeit im Folgenden nicht wiederholt.

#### Variablen zu Barrieren des Fahrradfahrens

Alle Teilnehmenden wurden wie folgt zu den Barrieren des Fahrradfahrens für das Zurücklegen alltäglicher Strecken befragt: „Im Folgenden sehen Sie verschiedene Gründe, die Menschen das alltägliche Zurücklegen von Wegstrecken mit dem Fahrrad erschweren können (wie zur Arbeit, zum Einkaufen, zu Freizeitaktivitäten). Welche Gründe treffen auf Sie zu und welche nicht?“ Es folgten 15 Aussagen zu individuellen und umweltbezogenen Barrieren des Radfahrens. Mehrfachnennung waren möglich. Zudem konnte ausgewählt werden: „Es gibt weitere Gründe, die mir das Zurücklegen meiner alltäglichen Wegstrecken mit dem Fahrrad erschweren.“ Die Antwortoptionen waren jeweils „Trifft zu“ und „Trifft nicht zu“. Der vollständige Fragebogen ist an anderer Stelle einsehbar [23].

Die Auswahl der abgefragten Barrieren basierte auf einer Literaturrecherche. Der Fragebogen wurde einem kognitiven Pretest unterzogen und anschließend überarbeitet [24].

### Statistische Methoden

Zur Schätzung der Häufigkeit des Fahrradfahrens und der Auswahlhäufigkeit der Barrieren wurden Prävalenzen mit den zugehörigen 95 %-Konfidenzintervallen (KI) berechnet. Zudem fand eine Stratifizierung nach soziodemografischen Faktoren statt. Für das Ranking wurden die Barrieren nach ihrer Auswahlhäufigkeit geordnet. Dies erfolgte für die gesamte Stichprobe (alle 15 Barrieren) sowie für 4 Untergruppen mit erhöhtem Bedarf an der Förderung des Fahrradfahrens (Frauen, Personen ab 75 Jahren, Personen mit niedriger Bildung und Personen in ländlichen Wohnregionen), für die jeweils die 5 am häufigsten ausgewählten Barrieren dargestellt wurden. Unterschiede in der Auswahlhäufigkeit der einzelnen Barrieren zwischen den soziodemografischen Gruppen wurden mittels multivariater logistischer Regressionsanalysen untersucht. Die berechneten Odds Ratios (OR) geben an, um welchen Faktor sich die Chancen (Odds) für die Auswahl einer Barriere gegenüber der jeweiligen Referenzgruppe unterscheiden. Als abhängige Variable wurde jeweils die Auswahl der betreffenden Barriere herangezogen; unabhängige Variablen waren Geschlechtsidentität, Alters- und Bildungsgruppe sowie Wohnregion. Zudem wurde für die Nutzung des Fahrrads als Form aktiver Mobilität adjustiert, um Unterschiede in der Fahrradnutzung zu berücksichtigen. Ein *p*-Wert < 0,05 wurde als statistisch signifikant angesehen.

Alle Analysen wurden mit Surveyverfahren in Stata/SE 17.0 (StataCorp, College Station, TX, USA) unter Berücksichtigung von Gewichtungsfaktoren durchgeführt. Durch die Anpassungsgewichtung wurden Abweichungen der Stichproben von der Bevölkerungsstruktur hinsichtlich Alter, Geschlecht, Bundesland, BIK-Raumtypen, Bildung und Haushaltsgröße korrigiert. Die Gewichtung erfolgte anhand der Bevölkerungsfortschreibung zum 31.12.2023 bzw. des Mikrozensus 2021 und dient der Verbesserung der Repräsentativität der Stichprobe.

## Ergebnisse

### Beschreibung der Stichprobe

Insgesamt nahmen 32.374 Personen an der Befragung teil, 54,8 % waren Frauen (Tab. 1). Die Altersspanne betrug 18 bis 100 Jahre und das mittlere Alter lag bei 54 Jahren. Personen, die der hohen Bildungsgruppe zugeordnet werden können, nahmen mit 34,9 % häufiger teil als Personen der niedrigen Bildungsgruppe (16,9 %).

**Tab. 1:** Stichprobenbeschreibung (*N* = 32.374)

Merkmal (Anzahl fehlende Werte)	*n*	Anteil in % (ungewichtet)	Anteil in % (gewichtet)
Geschlecht (100)			
Frauen	17.688	54,8	51,2
Männer	14.586	45,2	48,8
Altersgruppen (0)			
18–29 J.	4277	13,2	15,7
30–44 J.	6803	21,0	23,7
45–64 J.	10.950	33,8	33,3
65–79 J.	7483	23,1	18,6
80 J. und älter	2861	8,8	8,7
Bildungsgruppen (132)			
Niedrig	5454	16,9	32,2
Mittel	15.542	48,2	47,3
Hoch	11.246	34,9	20,4
Wohnregion (0)			
Ländlich	3722	11,5	10,5
Kleinstädtisch	8184	25,3	28,0
Mittelstädtisch	8483	26,2	30,3
Großstädtisch	11.985	37,0	31,2

Anmerkung: Aufgrund von Rundungen kann die Summe der Prozentanteile 100 % leicht über- oder unterschreiten

### Häufigkeit des Fahrradfahrens

Abgefragt wurde die Häufigkeit des Fahrradfahrens in einer typischen Woche bezogen auf die letzten 4 Wochen. 55,4 % der Erwachsenen fuhren nie mit dem Fahrrad, 13,9 % fuhren seltener als einmal pro Woche, 16,0 % fuhren an 1–3 Tagen und 14,7 % an mindestens 4 Tagen der Woche (Tab. 2).

**Tab. 2:** Häufigkeit des Fahrradfahrens zum Zurücklegen alltäglicher Wegstrecken (*n* = 31.804)

	Häufigkeit des Fahrradfahrens pro Woche (Anteil in % (95 %-KI))			
	Nie	< 1 Tag	1–3 Tage	Mind. 4 Tage
Gesamt	55,4 (53,9–56,9)	13,9 (13,2–14,5)	16,0 (15,3–16,7)	14,7 (13,8–15,7)
Geschlecht				
Frauen	60,6 (58,8–62,4)	12,5 (11,7–13,4)	13,4 (12,5–14,4)	13,5 (12,5–14,6)
Männer	50,0 (48,3–51,8)	15,3 (14,4–16,3)	18,7 (17,6–19,8)	16,0 (14,8–17,3)
Altersgruppen				
18–29 J.	56,8 (53,9–59,6)	15,1 (13,4–17,0)	13,8 (12,1–15,7)	14,3 (12,5–16,2)
30–44 J.	51,2 (49,0–53,5)	17,5 (16,1–19,0)	16,0 (14,6–17,5)	15,2 (13,7–16,9)
45–64 J.	52,3 (50,3–54,2)	13,8 (12,9–14,9)	17,9 (16,7–19,1)	16,0 (14,8–17,4)
65–79 J.	55,3 (53,0–57,6)	11,8 (10,8–13,0)	17,7 (16,2–19,2)	15,1 (13,8–16,6)
80 J. und älter	78,4 (75,8–80,8)	5,3 (4,0–7,0)	8,6 (7,3–10,2)	7,6 (6,3–9,2)
Bildungsgruppen				
Niedrig	62,0 (59,7–64,2)	12,7 (11,3–14,1)	12,8 (11,5–14,2)	12,5 (11,2–14,0)
Mittel	55,7 (54,1–57,3)	14,4 (13,6–15,2)	16,6 (15,6–17,6)	13,3 (12,3–14,3)
Hoch	44,6 (42,4–46,9)	14,8 (13,7–15,9)	19,6 (18,4–21,0)	21,0 (19,2–22,8)
Wohnregion				
Ländlich	62,6 (58,6–66,4)	14,2 (12,3–16,4)	15,2 (13,3–17,3)	8,0 (6,6–9,7)
Kleinstädtisch	56,1 (53,3–58,8)	14,2 (12,9–15,6)	17,0 (15,7–18,3)	12,8 (11,4–14,3)
Mittelstädtisch	55,7 (52,7–58,6)	14,1 (12,9–15,4)	15,8 (14,4–17,4)	14,4 (12,7–16,1)
Großstädtisch	52,2 (49,7–54,6)	13,2 (12,2–14,3)	15,5 (14,3–16,8)	19,1 (17,3–21,1)

*KI* Konfidenzintervall; aufgrund von Rundungen kann die Summe der Prozentanteile 100 % leicht über- oder unterschreiten

Frauen gaben häufiger an, nie Fahrrad gefahren zu sein, als Männer (60,6 % vs. 50,0 %) und Erwachsene ab 80 Jahren fuhren mit 78,4 % häufiger nie Fahrrad als die jüngeren Gruppen. Zudem zeigt sich bei der Fahrradnutzung ein Bildungsgradient: Von der hohen zur niedrigen Bildungsgruppe nahm der Anteil derjenigen zu, die nie Fahrrad fuhren (hohe Bildungsgruppe: 44,6 %; niedrige Bildungsgruppe: 62,0 %). Bei Personen aus ländlichen Regionen lag der Anteil derjenigen, die nie Fahrrad fuhren, mit 62,6 % höher als bei Personen aus städtischen Wohnregionen (Großstadt: 52,2 %).

### Barrieren des Fahrradfahrens

**Abb. 1:**
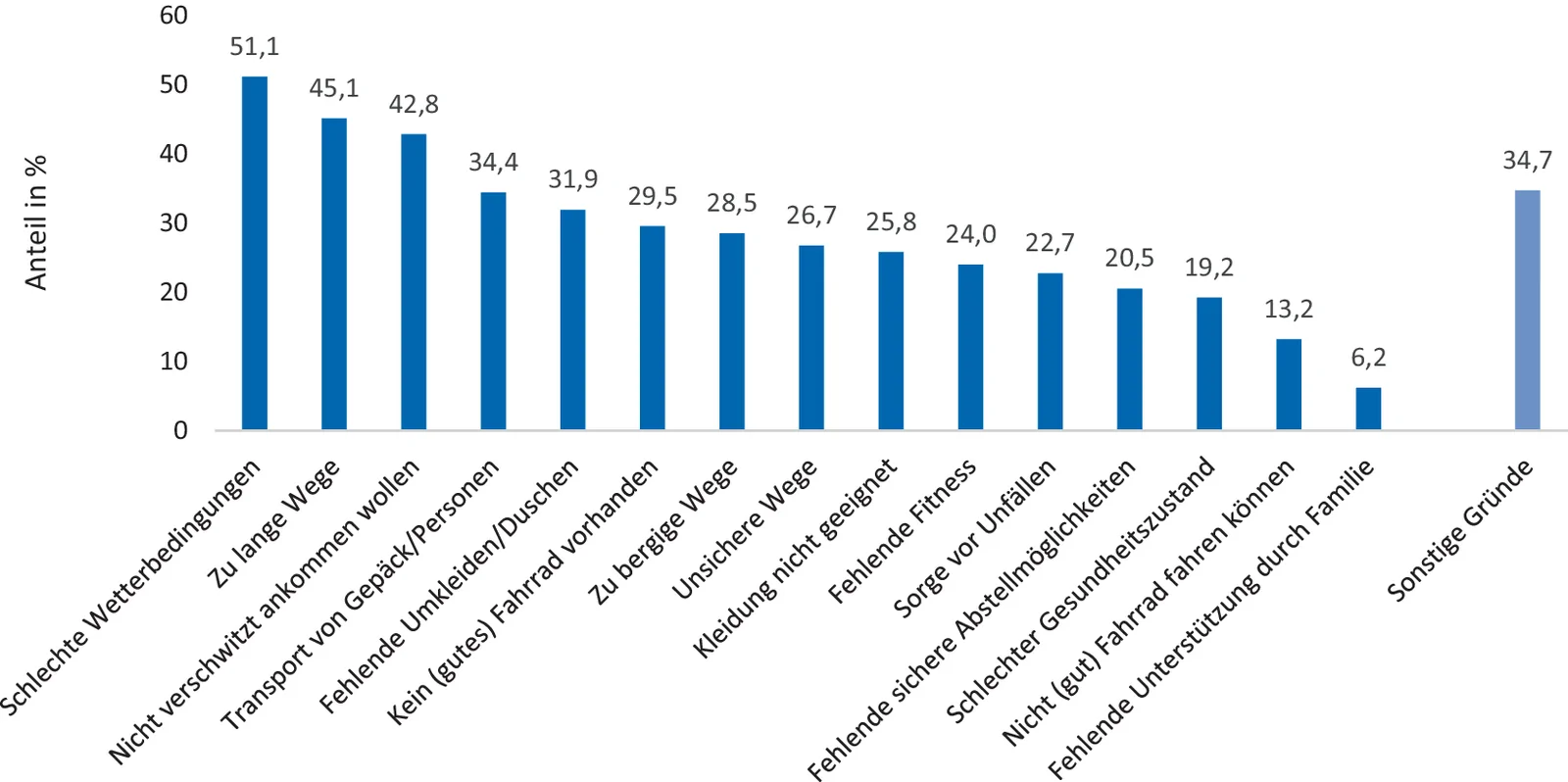
Häufigkeit der Auswahl der Barrieren in absteigender Reihenfolge (Fallzahlen zwischen *n* = 31.840 und *n* = 31.497)

Die Häufigkeitsverteilung der zutreffenden Barrieren (Ranking) ist in absteigender Reihenfolge in Abb. 1 dargestellt. Die 5 häufigsten Barrieren waren „schlechte Wetterbedingungen“ (51,1 %), „zu lange Wege“ (45,1 %), „nicht verschwitzt ankommen wollen“ (42,8 %), „Transport von Gepäck/Personen“ (34,4 %) und „fehlende Umkleiden/Duschen“ (31,9 %).

**Abb. 2:**
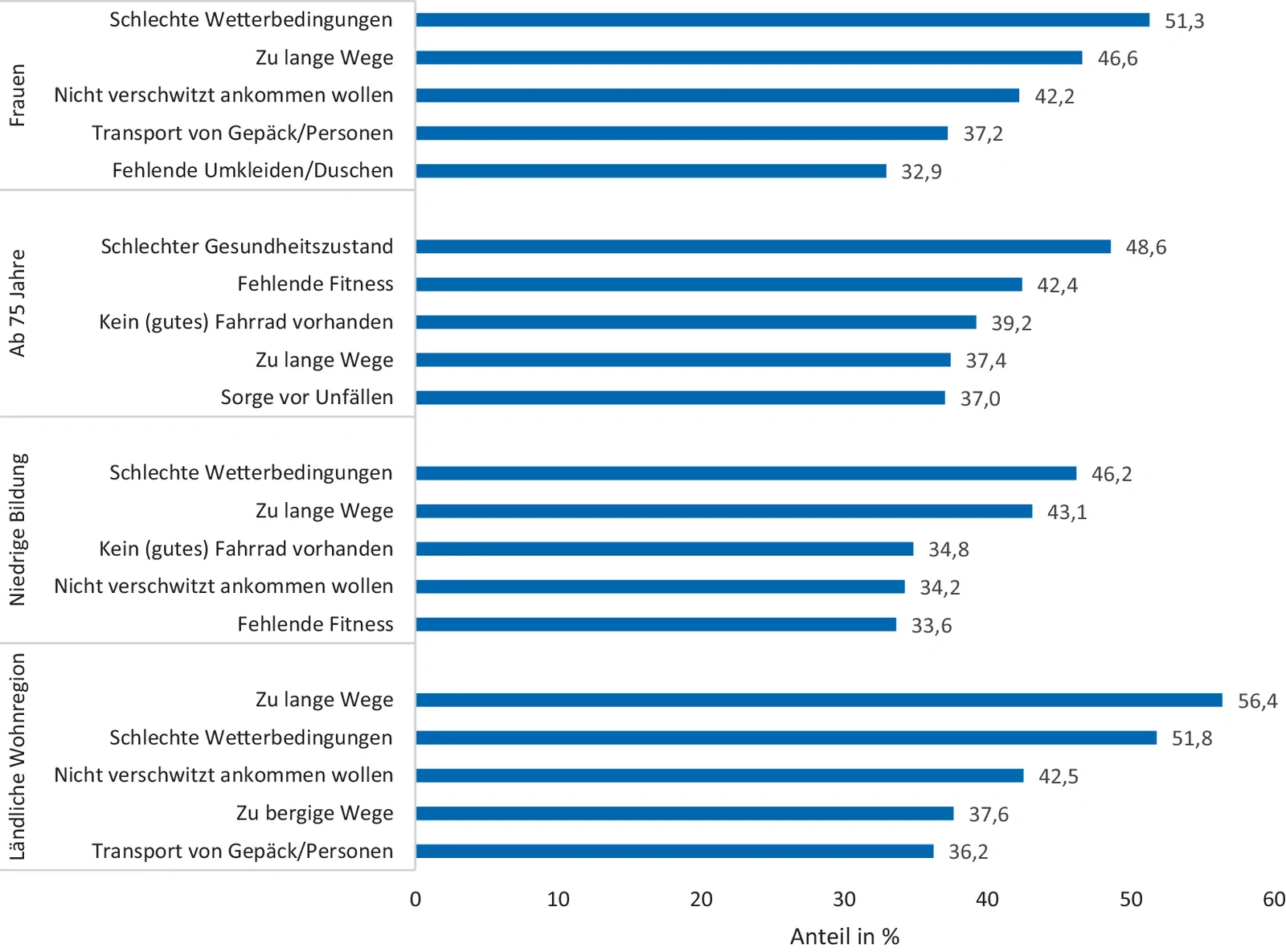
Die 5 am häufigsten genannten Barrien bei Bevölkerungsgruppen mit vermehrtem Förderbedarf der aktiven Mobilität

In den Bevölkerungsgruppen, die das Fahrrad seltener für alltägliche Wegstrecken nutzten und somit einen erhöhten Förderbedarf aufweisen, unterscheidet sich das Ranking der 5 häufigsten Barrieren teilweise von dem der Gesamtbevölkerung. Eine Ausnahme bilden Frauen, bei denen das Ranking dem der Gesamtbevölkerung entspricht (Abb. 2). Für Personen ab 75 Jahren sind die 5 häufigsten Barrieren des Fahrradfahrens „schlechter Gesundheitszustand“, „fehlende Fitness“, „kein (gutes) Fahrrad vorhanden“, „zu lange Wege“ und „Sorge vor Unfällen“. Im Unterschied zur Gesamtbevölkerung sind für Personen mit niedriger Bildung die Barrieren „kein (gutes) Fahrrad vorhanden“ und „fehlende Fitness“ Bestandteil der 5 häufigsten Barrieren und bei Personen aus einer ländlichen Wohnregion die Barriere „zu bergige Wege“.

### Soziodemografische Unterschiede

Die Häufigkeitsverteilung der Barrieren, stratifiziert nach soziodemografischen Faktoren, ist im zusätzlichen Onlinematerial (Tab. Z1) dargestellt. Nachfolgend werden die statistisch signifikanten Unterschiede in der Auswahlhäufigkeit der Barrieren nach soziodemografischen Variablen auf Grundlage der Ergebnisse der multivariaten Regressionsanalysen beschrieben (Tab. 3 und 4).

**Tab. 3:** Ergebnisse der multivariaten logistischen Regressionsanalysen

	Schlechte Wetterbedingungen	Zu lange Wege	Nicht verschwitzt ankommen wollen	Transport von Gepäck/Personen	Fehlende Umkleiden/Duschen	Kein (gutes) Fahrrad vorhanden	Zu bergige Wege	Unsichere Wege
	OR (95 %-KI)	OR (95 %-KI)	OR (95 %-KI)	OR (95 %-KI)	OR (95 %-KI)	OR (95 %-KI)	OR (95 %-KI)	OR (95 %-KI)
Geschlecht								
Frauen	1,06 (0,99–1,15)	1,10 (1,02–1,19)*	0,95 (0,88–1,03)	1,32 (1,22–1,43)*	1,11 (1,02–1,20)*	1,11 (1,02–1,22)*	1,34 (1,22–1,46)*	1,58 (1,46–1,71)*
Männer	Referenz	Referenz	Referenz	Referenz	Referenz	Referenz	Referenz	Referenz
Altersgruppen								
18–29 J.	Referenz	Referenz	Referenz	Referenz	Referenz	Referenz	Referenz	Referenz
30–44 J.	0,73 (0,64–0,84)*	0,92 (0,80–1,05)	0,70 (0,61–0,80)*	1,17 (1,02–1,35)*	0,92 (0,80–1,06)	0,70 (0,59–0,82)*	0,80 (0,68–0,94)*	0,97 (0,83–1,12)
45–64 J.	0,66 (0,57–0,75)*	0,85 (0,75–0,96)*	0,41 (0,36–0,47)*	0,76 (0,67–0,87)*	0,70 (0,62–0,80)*	0,48 (0,41–0,56)*	0,78 (0,68–0,90)*	0,81 (0,71–0,93)*
65–79 J.	0,44 (0,38–0,51)*	0,54 (0,47–0,62)*	0,17 (0,14–0,19)*	0,51 (0,44–0,59)*	0,32 (0,28–0,37)*	0,61 (0,52–0,72)*	0,84 (0,73–0,97)*	0,93 (0,81–1,08)
80 J. und älter	0,25 (0,21–0,30)*	0,50 (0,42–0,60)*	0,11 (0,09–0,14)*	0,23 (0,18–0,29)*	0,21 (0,17–0,26)*	0,73 (0,59–0,90)*	0,76 (0,62–0,92)*	0,87 (0,71–1,06)
Bildungsgruppen								
Niedrig	0,96 (0,86–1,08)	0,94 (0,84–1,04)	0,70 (0,62–0,79)*	0,69 (0,60–0,78)*	0,70 (0,62–0,80)*	1,52 (1,33–1,75)*	1,10 (0,94–1,27)	0,89 (0,78–1,02)
Mittel	1,07 (0,99–1,17)	1,01 (0,93–1,09)	0,84 (0,78–0,92)*	0,86 (0,79–0,94)*	0,96 (0,89–1,05)	1,16 (1,04–1,29)*	0,98 (0,88–1,09)	0,86 (0,78–0,94)*
Hoch	Referenz	Referenz	Referenz	Referenz	Referenz	Referenz	Referenz	Referenz
Wohnregion								
Ländlich	1,15 (0,99–1,32)	1,99 (1,72–2,30)*	1,02 (0,85–1,23)	1,35 (1,18–1,55)*	1,13 (0,95–1,35)	0,59 (0,50–0,70)*	2,11 (1,61–2,76)*	0,99 (0,79–1,23)
Kleinstädtisch	1,07 (0,96–1,19)	1,46 (1,31–1,62)*	0,96 (0,86–1,07)	1,37 (1,24–1,52)*	1,03 (0,92–1,16)	0,69 (0,60–0,78)*	1,67 (1,35–2,07)*	0,71 (0,62–0,82)*
Mittelstädtisch	1,13 (1,01–1,25)*	1,19 (1,07–1,32)*	0,95 (0,86–1,06)	1,23 (1,10–1,38)*	0,98 (0,87–1,10)	0,83 (0,74–0,94)*	1,54 (1,25–1,90)*	0,73 (0,63–0,84)*
Großstädtisch	Referenz	Referenz	Referenz	Referenz	Referenz	Referenz	Referenz	Referenz
*Fallzahlen*	*31.028*	*31.162*	*31.109*	*31.118*	*30.960*	*31.261*	*31.144*	31.172

Multivariate logistische Regression, abhängige Variable: „Barriere trifft zu“ (Referenz: Nein); adjustiert für die Häufigkeit des Fahrradfahrens; *OR* Odds Ratio, *KI* Konfidenzintervall, * statistisch signifikant mit *p* < 0,05

**Tab. 4:** Ergebnisse der multivariaten logistischen Regressionsanalysen (Fortsetzung Tab. 3)

	Kleidung nicht geeignet	Fehlende Fitness	Sorge vor Unfällen	Fehlende sichere Abstellmöglichkeiten	Schlechter Gesundheitszustand	Nicht (gut) Fahrrad fahren können	Fehlende Unterstützung durch Familie
	OR (95 %-KI)	OR (95 %-KI)	OR (95 %-KI)	OR (95 %-KI)	OR (95 %-KI)	OR (95 %-KI)	OR (95 %-KI)
Geschlecht							
Frauen	1,35 (1,24–1,48)*	1,38 (1,25–1,52)*	1,77 (1,60–1,95)*	1,11 (1,01–1,22)*	1,31 (1,18–1,46)*	2,04 (1,79–2,33)*	1,12 (0,94–1,34)
Männer	Referenz	Referenz	Referenz	Referenz	Referenz	Referenz	Referenz
Altersgruppen							
18–29 J.	Referenz	Referenz	Referenz	Referenz	Referenz	Referenz	Referenz
30–44 J.	0,78 (0,68–0,89)*	1,25 (1,02–1,53)*	0,97 (0,82–1,15)	0,76 (0,65–0,90)*	1,22 (0,92–1,63)	1,02 (0,75–1,39)	2,34 (1,45–3,77)*
45–64 J.	0,69 (0,61–0,78)*	1,47 (1,23–1,75)*	1,00 (0,86–1,15)	0,61 (0,53–0,71)*	2,09 (1,64–2,65)*	1,31 (0,99–1,74)	1,97 (1,25–3,10)*
65–79 J.	0,37 (0,32–0,42)*	1,70 (1,42–2,05)*	1,49 (1,27–1,73)*	0,52 (0,44–0,61)*	3,18 (2,51–4,01)*	3,04 (2,32–3,99)*	2,26 (1,41–3,62)*
80 J. und älter	0,28 (0,22–0,35)*	2,76 (2,23–3,40)*	1,96 (1,59–2,41)*	0,35 (0,27–0,44)*	8,42 (6,49–10,9)*	4,80 (3,59–6,41)*	2,38 (1,46–3,89)*
Bildungsgruppen							
Niedrig	0,63 (0,55–0,71)*	2,41 (2,07–2,81)*	1,15 (1,01–1,32)*	1,03 (0,89–1,20)	2,48 (2,13–2,89)*	2,03 (1,71–2,40)*	2,19 (1,71–2,81)*
Mittel	0,75 (0,68–0,82)*	1,60 (1,43–1,79)*	0,94 (0,85–1,04)	1,14 (1,03–1,26)*	1,40 (1,23–1,59)*	1,04 (0,90–1,20)	1,23 (0,99–1,53)
Hoch	Referenz	Referenz	Referenz	Referenz	Referenz	Referenz	Referenz
Wohnregion							
Ländlich	1,09 (0,91–1,30)	0,97 (0,80–1,17)	0,53 (0,44–0,65)*	0,82 (0,69–0,98)*	0,85 (0,70–1,03)	0,73 (0,59–0,90)*	0,84 (0,62–1,14)
Kleinstädtisch	0,99 (0,88–1,11)	0,85 (0,74–0,98)*	0,55 (0,48–0,64)*	0,83 (0,73–0,96)*	0,85 (0,74–0,99)*	0,74 (0,64–0,87)*	0,93 (0,73–1,20)
Mittelstädtisch	1,09 (0,98–1,23)	0,94 (0,82–1,07)	0,57 (0,50–0,65)*	0,89 (0,77–1,03)	0,79 (0,69–0,91)*	0,76 (0,66–0,89)*	0,93 (0,73–1,18)
Großstädtisch	Referenz	Referenz	Referenz	Referenz	Referenz	Referenz	Referenz
*Fallzahlen*	*31.065*	*31.096*	*31.169*	*31.036*	*31.274*	*31.214*	*31.039*

Multivariate logistische Regression, abhängige Variable: „Barriere trifft zu“ (Referenz: Nein); adjustiert für die Häufigkeit des Fahrradfahrens; *OR* Odds Ratio, *KI* Konfidenzintervall, * statistisch signifikant mit *p* < 0,05

Die Ergebnisse zeigen, dass für Frauen fast alle Barrieren häufiger zutreffen als für Männer. Ausnahmen sind „nicht verschwitzt ankommen wollen“, „schlechte Wetterbedingungen“ und „fehlende Unterstützung durch die Familie“. Insbesondere die Barrieren „nicht (gut) Fahrradfahren können“, „Sorge vor Unfällen“ und „unsichere Wege“ sind für Frauen häufiger zutreffend als für Männer.

Zudem zeigen die Ergebnisse Unterschiede zwischen den Altersgruppen. Folgende Barrieren treffen für ältere Erwachsene häufiger zu als für die jüngste Altersgruppe (18- bis 29-Jährige): „fehlende Fitness“, „fehlende Unterstützung durch die Familie“, „schlechter Gesundheitszustand“, „nicht (gut) Fahrradfahren können“, „Sorge vor Unfällen“ und „Transport von Gepäck oder Personen“. Besonders große Unterschiede zwischen der ältesten (ab 80 Jahren) und der jüngsten Altersgruppe (18–29 Jahre) zeigen sich bei den Barrieren „schlechter Gesundheitszustand“, „nicht (gut) Fahrradfahren können“ und „fehlende Fitness“.

Hingegen sind folgende Barrieren für ältere Erwachsene seltener zutreffend als für die jüngste Altersgruppe (18- bis 29-Jährige): „kein (gutes) Fahrrad vorhanden“, „zu bergige Wege“, „nicht verschwitzt ankommen wollen“, „Kleidung nicht geeignet“, „schlechte Wetterbedingungen“, „fehlende sichere Abstellmöglichkeiten“, „fehlende Umkleiden/Duschmöglichkeiten“, „zu lange Wege“, „Transport von Gepäck oder Personen“ und „unsichere Wege“. Besonders große Unterschiede zwischen der ältesten und der jüngsten Altersgruppe zeigen sich bei den Barrieren „nicht verschwitzt ankommen wollen“, „fehlende Umkleiden/Duschmöglichkeiten“ und „Transport von Gepäck oder Personen“.

Für Personen der niedrigen und bzw. oder mittleren Bildungsgruppen sind folgende Barrieren häufiger zutreffend als für Personen der hohen Bildungsgruppe: „schlechter Gesundheitszustand“, „kein (gutes) Fahrrad vorhanden“, „fehlende Fitness“, „nicht (gut) Fahrradfahren können“, „Sorge vor Unfällen“, „fehlende Unterstützung durch die Familie“ und „fehlende sichere Abstellmöglichkeiten“. Besonders große Unterschiede zwischen der niedrigen und der hohen Bildungsgruppe bestehen bei den Barrieren „schlechter Gesundheitszustand“, „fehlende Fitness“ und „fehlende Unterstützung durch die Familie“.

Hingegen wurden folgende Barrieren von Personen der niedrigen und/oder mittleren Bildungsgruppe seltener als zutreffend ausgewählt als von Personen der hohen Bildungsgruppe: „nicht verschwitzt ankommen wollen“, „Transport von Gepäck oder Personen“, „Kleidung nicht geeignet“, „unsichere Wege“ und „fehlende Umkleiden/Duschmöglichkeiten“. Die Effektstärken unterscheiden sich zwischen den aufgeführten Barrieren nur geringfügig.

Für Personen, die in einer ländlichen, klein- oder mittelstädtischen Wohnregion wohnen, treffen folgende Barrieren häufiger zu als für Personen aus der Großstadt: „zu lange Wege“, „zu bergige Wege“, „Transport von Gepäck oder Personen“ und „schlechte Wetterbedingungen“. Größere Unterschiede zwischen Personen aus ländlichen Wohnregionen und der Großstadt bestehen vor allem bei „zu bergige Wege“ und „zu lange Wege“.

Für Personen aus einer ländlichen, klein- oder mittelstädtischen Region treffen folgende Barrieren seltener zu als für Personen aus der Großstadt: „nicht (gut) Fahrrad fahren können“, „kein (gutes) Fahrrad vorhanden“, „Sorge vor Unfällen“, „fehlende sichere Abstellmöglichkeiten“, „unsichere Wege“, „schlechter Gesundheitszustand“ und „fehlende Fitness“. Besonders große Unterschiede zwischen Personen aus einer ländlichen Wohnregion und der Großstadt bestanden bei „Sorge vor Unfällen“ und „kein (gutes) Fahrrad vorhanden“.

## Diskussion

Die Studienergebnisse zeigen, dass sich viele der am häufigsten ausgewählten Barrieren auf die natürliche oder bebaute Umwelt beziehen, beispielsweise schlechte Wetterbedingungen, zu lange Wege und fehlende Dusch- und Umkleidemöglichkeiten. Aber auch individuelle Barrieren, wie nicht verschwitzt am Zielort ankommen zu wollen oder der Transport von Gepäck oder Personen, wurden von mindestens jeder dritten Person als zutreffend gekennzeichnet.

Die am häufigsten zutreffende Barriere ist „schlechte Wetterbedingungen“, was sowohl Regen, Schnee und kühle Temperaturen sowie Hitze beinhalten kann. Da die Wetterbedingungen nicht veränderbar sind, benötigt es eine Anpassung an widrige Wetterverhältnisse sowohl auf der Wegstrecke mit entsprechender Kleidung und Wetterschutz als auch am Zielort. Die Bereitstellung von Dusch- und Umkleidemöglichkeiten sowie von Schränken/Garderoben zur Lagerung von Kleidung am Zielort kann dazu beitragen, mit widrigen Wetterverhältnissen besser umgehen zu können [25]. Verdeutlicht wird die Bedeutung von Dusch- und Umkleidemöglichkeiten auch dadurch, dass nahezu jede dritte Person in der vorliegenden Studie angab, dass fehlende Umkleide- und Duschmöglichkeiten an Zielorten hinderlich für das Fahrradfahren sind. Arbeitgeber können durch die Bereitstellung dieser Räumlichkeiten und weitere Maßnahmen die Fahrradnutzung auf dem Arbeitsweg fördern.

Die am zweithäufigsten zutreffende Barriere ist „zu lange Wege“. Diese Barriere wurde vermehrt in ländlichen Regionen ausgewählt, aber auch für rund 40 % der Personen aus Großstädten sind zu lange Wege hinderlich. Langfristig kann eine veränderte Stadtplanung nach dem Leitbild „Stadt der Viertelstunde“ dazu beitragen, dass möglichst viele Orte des alltäglichen Lebens innerhalb von 15 min mit dem Fahrrad oder zu Fuß zu erreichen sind (Nutzungsmischung; [25, 26]). Für den ländlichen Raum werden mit dem Konzept „30-Minuten-Land“ ähnliche Ansätze diskutiert [27]. Des Weiteren trägt die Kombination aus Fahrradfahren und der Nutzung des öffentlichen Personennahverkehrs (ÖPNV) dazu bei, dass zumindest Teilstrecken mit dem Fahrrad zurückgelegt werden [28]. Förderlich wirken sich diesbezüglich sichere Abstellanlagen an Bahnhöfen, ein attraktives System zur Mitnahme des Fahrrads im ÖPNV sowie Fahrrad-Leihsysteme aus [25]. Darüber hinaus können bei längeren Wegen Pedelecs helfen, diese mit dem Fahrrad zurückzulegen. Finanzielle Förderungen zur Anschaffung eines Pedelecs und attraktive Leihsysteme könnten auch Personen mit geringeren finanziellen Ressourcen die Nutzung von Pedelecs ermöglichen [25, 29]. In Bezug auf Lastenräder könnten eine finanzielle Förderung und gute Verleihsysteme auch dabei unterstützen, Gepäck und zumindest jüngere Kinder zu transportieren, was ebenfalls häufig als Barriere angegeben wurde.

Die am dritthäufigsten zutreffende Barriere ist, nicht verschwitzt am Zielort ankommen zu wollen. Die bereits diskutierte Maßnahme, Umkleide- und Duschmöglichkeiten auszubauen, kann diesbezüglich ebenfalls förderlich sein. Zudem kann eine Steigerung der körperlichen Leistungsfähigkeit durch regelmäßiges Fahrradfahren dazu beitragen, dass das aktive Zurücklegen alltäglicher Wegstrecken als weniger anstrengend empfunden wird. In der vorliegenden Studie gab jede 4. Person an, dass eine nicht ausreichende Fitness sie daran hindert, Wege mit dem Fahrrad zurückzulegen. Maßnahmen zur Förderung der bewegungsbezogenen Gesundheitskompetenz können dazu beitragen, dass Personen lernen, ihre körperliche Leistungsfähigkeit sukzessiv zu steigern [30].

Frauen, ältere Erwachsene, Personen mit formal niedrigerer Bildung und Personen mit Wohnort in einer ländlichen Region nutzen seltener das Fahrrad, um alltägliche Wege zurückzulegen, und haben somit einen erhöhten Förderbedarf. Die vorliegenden Ergebnisse zeigen, dass sich die Relevanz der einzelnen Barrieren des Fahrradfahrens zwischen Frauen und Männern, den Alters- und Bildungsgruppen sowie einer ländlichen und städtischen Wohnregion teilweise deutlich unterscheidet.

Während die Reihenfolge der am häufigsten ausgewählten Barrieren zwischen Frauen und Männern die gleiche ist, wählten Frauen 12 der 15 Barrieren signifikant häufiger aus als Männer. Insbesondere die Barrieren „Sorge vor Unfällen“, „unsichere Wege“ und „nicht (gut) Fahrrad fahren können“ wurden von Frauen häufiger ausgewählt. Studien zeigen, dass fehlende Sicherheit für Frauen ein Hinderungsgrund für das Fahrradfahren ist und Maßnahmen wie der Ausbau baulich vom motorisierten Verkehr getrennter Fahrradwege zu einer Steigerung des Radverkehrsanteils von Frauen beitragen kann [19, 31]. Die Vielzahl der häufiger von Frauen als von Männern ausgewählten Barrieren zeigt zudem, dass die Hinderungsgründe von Frauen komplex sind.

Mit zunehmendem Alter nimmt erwartungsgemäß die Relevanz der gesundheits- und fitnessbezogenen Barrieren zu, wodurch ein schlechter Gesundheitszustand bei Personen ab 75 Jahren die häufigste Barriere ist. Insbesondere bei hochaltrigen Personen können altersbedingte körperliche Einschränkungen das Fahrradfahren im Straßenverkehr erschweren. Auch die Sorge vor Unfällen, die als Barriere ab 65 Jahren vermehrt ausgewählt wurde, kann in Zusammenhang mit der Zunahme von körperlichen Einschränkungen im Alter stehen. Der Ausbau einer sicheren Fahrradinfrastruktur und eine reduzierte Geschwindigkeit des motorisierten Verkehrs können dazu beitragen, dass sich auch Personen mit körperlichen oder kognitiven Einschränkungen zutrauen, alltägliche Wege mit dem Fahrrad zurückzulegen [25, 32].

Personen mit formal niedriger Bildung gaben einen schlechten Gesundheitszustand und fehlende Fitness häufiger als Barrieren an. Die höhere Relevanz der Barriere „fehlende Fitness“ in dieser Gruppe lässt sich durch das niedrigere körperliche Aktivitätsniveau im Vergleich zu höher gebildeten Personen erklären [33]. Zudem wurde vermehrt genannt, dass kein (gutes) Fahrrad vorhanden ist. Finanzielle Förderungen für den Erwerb von Fahrrädern, Reparaturangebote sowie bedarfsgerechte Verleihsysteme können den Zugang zum Fahrradfahren für Personen aus benachteiligten sozioökonomischen Verhältnissen erleichtern. Darüber hinaus können zielgruppenorientierte Kampagnen und aufklärende Maßnahmen zu den gesundheitlichen Vorteilen des Fahrradfahrens den Einstieg fördern.

Für Personen aus ländlichen Wohnregionen sind die Barrieren „Wege zu lang“ und „Wege zu bergig“ häufiger zutreffend als für Personen aus städtischen Regionen. Die bereits beschriebenen Maßnahmen, wie eine vermehrte Nutzungsmischung in der Wohnregion, attraktive Kombinationsmöglichkeiten von Fahrrad und ÖPNV, können Personen in ländlichen Regionen unterstützten, vermehrt Wege mit dem Fahrrad zurückzulegen [34].

Die Ergebnisse des „Fahrrad-Monitors 2025“ bestätigen für Personen, die das Fahrrad nie als Verkehrsmittel nutzen, dass Aspekte wie das Wetter, zu weite Strecken, der Transport von Dingen sowie Sicherheitsaspekte relevante Hinderungsgründe sein können [19]. Die Ergebnisse einer weiteren in Deutschland durchgeführten Studie aus dem Jahr 2024 weisen zudem darauf hin, dass eine fehlende Sozialisation mit dem Fahrrad als Mobilitätsform ein wichtiger Hinderungsgrund sein kann [35]. Eine unterschiedliche Mobilitätsozialisation in Bezug auf das Fahrradfahren kann auch eine Erklärung dafür sein, dass in der deutschen Bevölkerung mehr als die Hälfte der Personen Wetterbedingungen für das Fahrradfahren als hinderlich empfinden, während das Wetter in Fahrradstädten wie Amsterdam oder Kopenhagen kein relevanter Hinderungsgrund zu sein scheint.

### Stärken und Limitationen

Die Ergebnisse der Studie ermöglichen repräsentative Aussagen zu den subjektiven Barrieren des Fahrradfahrens für die erwachsene Bevölkerung. Zudem konnten durch die nach Geschlechts‑, Alters- und Bildungsgruppen sowie der Wohnregion differenzierten Analysen Unterschiede in der Wahrnehmung von Barrieren zwischen soziodemografischen Gruppen aufgezeigt werden. Folgende Limitationen sollten bei der Interpretation der Ergebnisse berücksichtigt werden: Aufgrund begrenzter zeitlicher Ressourcen im Rahmen der Befragung konnte nur eine gewisse Anzahl an Barrieren quantitativ erfasst werden, sodass weitere mögliche Barrieren, wie soziale Normen und rücksichtsloses Verhalten von Autofahrenden [36], hier unberücksichtigt bleiben. Das zeigt sich auch daran, dass jede dritte Person die Kategorie „sonstige Gründe“ angekreuzt hat. Zudem können sich Barrieren untereinander bedingen. So haben beispielsweise schlechte Wetterbedingungen einen höheren Stellenwert, wenn die Wege lang sind, was in der vorliegenden Analyse nicht berücksichtigt wurde. Des Weiteren kann es durch die subjektive Erfassung der Barrieren zu sozial erwünschtem Antwortverhalten kommen, wodurch gegebenenfalls Barrieren, wie fehlende Fitness oder nicht gut Fahrrad fahren können, seltener ausgewählt wurden.

## Fazit

Die Ergebnisse verdeutlichen, dass die Hinderungsgründe für das Zurücklegen von Wegstrecken mit dem Fahrrad vielfältig sind und sowohl die umweltbezogene als auch die individuelle Ebene betreffen. Dementsprechend sollten Maßnahmen zur Förderung des Fahrradfahrens mehrere Komponenten berücksichtigen [25, 29]. Derzeit gibt es in Deutschland bereits Bestrebungen, die Rahmenbedingungen für das Fahrradfahren zu verbessern. Ein Teil der beschriebenen Maßnahmen ist Bestandteil des Nationalen Radverkehrsplans 3.0 der Bundesregierung und befindet sich bereits in der Umsetzung [37]. Jedoch fehlt es bislang an einer flächendeckenden Verwirklichung der vorgesehenen Maßnahmen und insbesondere am konsequenten Ausbau eines einladenden Radverkehrsnetzes. Fördermaßnahmen in unterschiedlichen Bereichen, wie Verkehr, Gesundheit und Städtebau, sowie eine verstärkte Zusammenarbeit dieser Sektoren auf kommunaler, Landes- und Bundesebene sind weiterhin erforderlich, um das Fahrradfahren für die gesamte Bevölkerung zu einer attraktiven Option der alltäglichen Fortbewegung zu etablieren und damit maßgeblich zur Gesundheitsförderung beizutragen.

## Supplementary Information

Onlinematerial: Prävalenzen der Auswahl der Barrieren stratifiziert nach soziodemografischen Merkmalen.

## Data Availability

Für die den Ergebnissen zugrunde liegenden Daten bestehen Zugangsbeschränkungen. Die Daten sind bei begründetem Forschungsinteresse im Rahmen einer erweiterten Datennutzung über das Forschungsdatenzentrum (FDZ) des Robert Koch-Instituts zugänglich. Anfragen können per E‑Mail an fdz@rki.de gestellt werden. Weitere Informationen finden sich auf der folgenden Webseite: www.rki.de/fdz.
