# Supplementary material for: Barriers to cycling for transportation: findings from the population-based survey “Health in Germany”
Source: Bundesgesundheitsblatt Gesundheitsforschung Gesundheitsschutz. 2026 Oct 8;69(11):1326–35. [Article in German] doi: 10.1007/s00103-026-04311-w (PMC13714075; doi:10.1007/s00103-026-04311-w)
Supplement: Supplementary file 1 [file 103_2026_4311_MOESM1_ESM.pdf]

Onlinematerial Tabelle Z1 *Prävalenzen der Auswahl der Barrieren stratifiziert nach soziodemografischen Merkmalen*

	Schlechte Wetterbedingungen<br>% (95% KI)	Zu lange Wege<br>% (95% KI)	Nicht verschwitzt ankommen wollen<br>% (95% KI)	Transport von Gepäck/Personen<br>% (95% KI)	Fehlende Umkleiden/ Duschen<br>% (95% KI)	Kein (gutes) Fahrrad vorhanden<br>% (95% KI)	Zu bergige Wege<br>% (95% KI)
Fallzahl	*31.571*	*31.710*	*31.652*	*31.664*	*31.497*	*31.811*	*31.694*
Geschlecht							
Frauen	51,3 (50,0-52,6)	46,6 (45,3-48,0)	42,2 (40,9-43,4)	37,2 (35,9-38,6)	37,2 (35,9-38,6)	32,2 (30,8-33,7)	32,0 (30,0-34,1)
Männer	50,9 (49,5-52,3)	43,6 (42,0-45,1)	43,5 (42,0-44,9)	31,5 (30,3-32,8)	31,5 (30,3-32,8)	26,6 (25,3-27,9)	24,9 (23,0-26,9)
Altersgruppen							
18-29 J.	62,4 (59,6-65,1)	50,6 (47,8-53,3)	63,5 (60,7-66,2)	40,0 (37,4-42,7)	42,2 (39,4-45,0)	37,6 (35,1-40,3)	31,0 (28,0-34,1)
30-44 J.	55,4 (53,4-57,3)	48,1 (45,9-50,2)	54,9 (53,0-56,9)	44,5 (42,4-46,6)	39,8 (37,8-41,8)	28,8 (26,8-30,9)	26,6 (24,1-29,1)
45-64 J.	52,6 (51,2-54,1)	47,0 (45,4-48,6)	41,3 (39,9-42,8)	34,2 (32,7-35,7)	33,0 (31,5-34,5)	23,3 (21,7-24,9)	27,4 (25,2-29,6)
65-79 J.	42,1 (40,4-43,7)	36,4 (34,6-38,3)	22,3 (20,8-23,8)	25,4 (23,8-27,0)	18,2 (16,9-19,4)	29,2 (27,3-31,2)	29,6 (27,5-31,9)
80 J. und älter	28,9 (26,1-31,8)	37,5 (34,5-40,6)	17,9 (15,6-20,4)	14,4 (12,2-16,9)	13,4 (11,6-15,4)	42,5 (39,2-45,9)	31,8 (28,8-35,0)
Bildungsgruppen							
niedrig	46,2 (44,0-48,3)	43,1 (41,2-45,1)	34,2 (32,1-36,4)	27,8 (25,9-29,8)	24,3 (22,5-26,2)	34,8 (32,8-36,8)	31,4 (29,0-33,9)
mittel	54,4 (53,1-55,7)	47,2 (45,8-48,6)	46,4 (45,1-47,7)	37,3 (36,0-38,7)	35,9 (34,6-37,2)	28,8 (27,5-30,2)	28,2 (26,1-30,3)
hoch	51,4 (49,8-52,9)	43,5 (41,7-45,4)	47,6 (46,1-49,2)	38,4 (36,8-40,1)	34,4 (33,0-35,9)	22,9 (21,4-24,5)	24,7 (22,4-27,1)
Wohnregion							
Ländlich	51,8 (48,8-54,8)	56,4 (53,4-59,3)	42,5 (38,7-46,4)	36,2 (33,7-38,7)	33,4 (30,1-36,9)	26,5 (23,5-29,7)	37,6 (32,2-43,3)
Kleinstädtisch	50,7 (49,1-52,3)	48,4 (46,4-50,4)	41,5 (39,7-43,3)	36,8 (35,1-38,6)	31,6 (30,0-33,3)	27,0 (25,1-29,1)	31,3 (28,0-34,9)
Mittelstädtisch	52,0 (50,2-53,7)	43,6 (41,7-45,6)	41,7 (39,8-43,7)	34,4 (32,5-36,3)	30,8 (29,0-32,7)	30,4 (28,4-32,5)	30,1 (26,8-33,6)
Großstädtisch	50,4 (48,5-52,3)	39,8 (38,2-41,5)	45,2 (43,6-46,9)	31,7 (30,3-33,2)	32,6 (30,8-34,4)	31,9 (30,0-33,8)	21,4 (19,0-23,9)

Onlinematerial Fortsetzung Tabelle Z1

	Unsichere Wege<br>% (95% KI)	Kleidung nicht geeignet<br>% (95% KI)	Fehlende Fitness<br>% (95% KI)	Sorge vor Unfällen<br>% (95% KI)	Fehlende sichere Abstell-möglichkeiten<br>% (95% KI)	Schlechter Gesundheits-zustand<br>% (95% KI)	Nicht (gut) Fahrrad fahren können<br>% (95% KI)	Fehlende Unterstützung durch Familie<br>% (95% KI)
Fallzahl	*31.723*	*31.605*	*31.639*	*31.716*	*31.576*	*31.840*	*31.765*	*31.581*
Geschlecht								
Frauen	31,4 (29,9-32,9)	28,6 (27,4-29,8)	27,9 (26,7-29,2)	28,0 (26,6-29,4)	21,4 (20,3-22,5)	22,3 (21,1-23,5)	17,7 (16,6-18,8)	6,7 (6,0-7,5)
Männer	21,8 (20,5-23,1)	22,9 (21,7-24,1)	19,9 (18,7-21,1)	17,0 (15,8-18,3)	19,6 (18,4-20,8)	15,9 (14,9-17,0)	8,5 (7,7-9,3)	5,6 (4,9-6,3)
Altersgruppen								
18-29 J.	28,6 (26,1-31,3)	34,4 (32,2-36,8)	17,7 (15,8-19,9)	20,8 (18,8-23,1)	28,5 (26,1-31,0)	9,7 (8,1-11,6)	7,6 (6,2-9,3)	3,2 (2,1-4,6)
30-44 J.	27,6 (25,8-29,5)	30,0 (28,1-31,9)	19,1 (17,4-21,0)	19,3 (17,5-21,1)	22,6 (20,9-24,5)	10,4 (9,1-12,0)	7,2 (6,2-8,5)	6,4 (5,3-7,7)
45-64 J.	24,2 (22,8-25,8)	26,6 (25,3-28,0)	23,2 (21,8-24,6)	19,5 (18,2-21,0)	19,2 (17,9-20,5)	17,4 (16,1-18,8)	9,7 (8,7-10,8)	5,9 (5,1-6,8)
65-79 J.	27,1 (25,3-29,1)	16,0 (14,7-17,4)	27,8 (26,2-29,5)	27,3 (25,6-29,1)	16,4 (15,0-18,0)	26,3 (24,8-28,0)	21,2 (19,5-22,9)	7,3 (6,4-8,4)
80 J. und älter	29,7 (26,7-32,8)	14,6 (12,5-17,1)	46,4 (43,5-49,3)	39,4 (36,5-42,5)	12,9 (10,9-15,2)	55,3 (52,3-58,2)	39,0 (35,9-42,2)	10,0 (8,4-12,0)
Bildungsgruppen								
niedrig	26,9 (24,9-29,0)	20,6 (19,0-22,4)	33,6 (31,6-35,6)	26,7 (24,8-28,8)	18,4 (16,7-20,2)	30,8 (28,9-32,7)	21,9 (20,2-23,7)	9,1 (7,9-10,5)
mittel	26,2 (24,8-27,6)	27,2 (25,9-28,4)	21,9 (20,9-23,0)	20,7 (19,5-22,0)	22,4 (21,3-23,6)	14,9 (14,1-15,9)	9,5 (8,8-10,3)	5,0 (4,4-5,6)
hoch	27,7 (25,9-29,5)	30,3 (28,9-31,8)	13,9 (12,9-15,0)	20,7 (19,1-22,3)	19,4 (18,1-20,7)	10,7 (9,7-11,6)	7,9 (7,2-8,8)	4,1 (3,4-4,8)
Wohnregion								
Ländlich	30,8 (26,5-35,4)	26,1 (23,1-29,4)	27,1 (24,1-30,3)	20,4 (17,5-23,5)	19,0 (16,9-21,4)	21,2 (18,4-24,3)	13,8 (11,4-16,7)	6,0 (4,8-7,5)
Kleinstädtisch	23,7 (21,9-25,7)	24,5 (22,9-26,2)	23,2 (21,5-25,0)	19,7 (18,1-21,4)	19,1 (17,6-20,6)	19,4 (17,9-21,1)	12,5 (11,4-13,7)	6,3 (5,4-7,4)
Mittelstädtisch	24,5 (22,5-26,6)	26,6 (24,8-28,5)	24,7 (23,2-26,3)	20,5 (18,8-22,2)	20,3 (18,5-22,2)	18,6 (17,3-20,0)	13,0 (11,8-14,4)	6,2 (5,4-7,2)
Großstädtisch	30,1 (28,2-32,2)	26,1 (24,9-27,4)	22,9 (21,2-24,8)	28,2 (26,3-30,3)	22,5 (20,9-24,2)	18,8 (17,2-20,4)	13,7 (12,5-14,9)	6,0 (5,1-7,1)

.
